# Epithelioid peritoneal mesothelioma: a hybrid phenotype within a mesenchymal-epithelial/epithelial-mesenchymal transition framework

**DOI:** 10.18632/oncotarget.12262

**Published:** 2016-09-26

**Authors:** Fabio Bozzi, Silvia Brich, Gian Paolo Dagrada, Tiziana Negri, Elena Conca, Barbara Cortelazzi, Antonino Belfiore, Federica Perrone, Ambra Vittoria Gualeni, Annunziata Gloghini, Antonello Cabras, Monica Brenca, Roberta Maestro, Nadia Zaffaroni, Paolo Casali, Rossella Bertulli, Marcello Deraco, Silvana Pilotti

**Affiliations:** ^1^ Laboratory of Experimental Molecular Pathology, Department of Pathology and Laboratory Medicine, Fondazione IRCCS Istituto Nazionale dei Tumori, Milan, Italy; ^2^ Department of Diagnostic Pathology and Laboratory Medicine, Fondazione IRCCS Istituto Nazionale dei Tumori, Milan, Italy; ^3^ Molecular Pharmacology Unit, Department of Experimental Oncology and Molecular Medicine, Fondazione IRCCS Istituto Nazionale dei Tumori, Milan, Italy; ^4^ Adult Mesenchymal Tumor Medical Oncology Unit, Department of Cancer Medicine, Fondazione IRCCS Istituto Nazionale dei Tumori, Milan, Italy; ^5^ Department of Surgery, Fondazione IRCCS Istituto Nazionale dei Tumori, Milan, Italy; ^6^ Experimental Oncology 1, Centro di Riferimento Oncologico, CRO Aviano National Cancer Institute, Aviano, Italy; ^7^ MOSE-DEA University of Trieste, Trieste, Italy

**Keywords:** malignant peritoneal mesothelioma, MET, MErT

## Abstract

The aim of this study was to reconsider the biological characteristics of epithelioid malignant peritoneal mesothelioma (E-MpM) in the light of new concepts about epithelial mesenchymal transition and mesenchymal epithelial reverse transition (EMT/MErT) and the role of epigenetic reprogramming in this context. To this end we profiled surgical specimens and derived cells cultures by a number of complementary approaches i.e. immunohistochemistry, immunofluorescence, in situ hybridization, biochemistry, pluripotent stem cell arrays, treatments with cytokines, growth factors and specific inhibitors.

The analyses of the surgical specimens showed that i) EZH2 is expressed throughout the spectrum of MpM, ii) that E-MpM (including the high-grade undifferentiated form) are characterised by c-MYC and miRNA 17-5p expression, and iii) that progression to sarcomatoid MpM is dictated by EMT regulators. They also showed that E-MpM expressed c-MET and are enriched in E- and P-cadherins- and VEGFR2-expressing CSCs, thus strongly supporting a role for MErT reprogramming in endowing E-MpM tumour cells with stemness and plasticity, and hence with a drug resistant phenotype. The cell culture-based experiments confirmed the stemness traits and plasticity of E-MpM, and support the view that EZH2 is a druggable target in this tumor.

## INTRODUCTION

Epithelial-mesenchymal transition (EMT) and mesenchymal-epithelial reverse transition (MErT) play crucial roles in embryogenesis, wound healing and cancer progression/metastases [1]. They are highly dynamic and tightly controlled processes that involve transcription factors, chromatin reorganisation factors, surface receptors and adhesion molecules [2, 3]. The activation of EMT makes cancer cells highly plastic [4], and enable non-cancer stem cells to acquire the traits of cancer stem cells (CSCs) [5].

Two recent studies [6, 7] have challenged the field of EMT research by introducing the concept of hybrid EMT. Both studies suggest that cells that undergo partial EMT, i.e. cells co-expressing epithelial and mesenchymal markers, are more plastic and have an high degree of stemness sustained by cell-cell adhesion compared to fully transited cells. This new view implies that cells with a hybrid phenotype can exploit a wider repertoire of survival strategies thus making it easier to evade cell death and anti-cancer treatments.

Malignant peritoneal mesothelioma (MpM) is a heterogeneous, aggressive tumour mainly caused by exposure to asbestos. It has been shown that all three types of asbestos are the most potent micro-enviromental inducers of MErT by means of an epigenetic process [8] or direct interaction with mesothelial cells [9]. MpMs express markers such as calretinin, cytokeratin 5/6 and podoplanin [10, 11], which are routinely used for diagnosis, and markers that have been more recently described such as VEGFR2 [12] and c-MET [13], or more widely explored in pre-clinical settings such as E-cadherin [14] and c-MYC [15].

MErT can be initiated by many signalling effectors [16], including the hepatocyte growth factor/scatter factor (HGF/SF)-mediated activation of c-MET in mesenchymal cells [17, 18]. It has been reported that, in addition to mediating intracellular cohesion, E-cadherin participates in regulating stemness [19–21], and the same is true for c-MYC [22, 23]. The recently described co-expression of E- and P-cadherin is particularly interesting as it has been reported to be a marker of stemness [24] or related to poor prognosis [25], and can be seen in the hybrid state of EMT/MErT [6, 7, 24].

It has been suggested that EMT is a significant morphological feature of malignant mesothelioma [25], and that aberrant polycomb-group (PcG) protein expression contributes to the pathogenesis of MpM [26]. We here propose a model of MpM in which the epithelioid variant (E-MpM), the most frequently encountered, at this site has the characteristics of MErT, and displays features of reprogramming with involvement of c-MYC, E- and P-cadherins and VEGFR2 as CSC regulators. E-MpMs may progress toward the undifferentiated high-grade (HG-MpM) variant or a sarcomatous MpM (S-MpM), which is characterised by a change in immunophenotypical make up from the pluripotent OMSK (OCT4, c-MYC SOX2, and KLF4) marker c-MYC to the TWIST and SLUG EMT regulators. The PcG protein EZH2, which acts as a catalytic subunit in polycomb repressive complex 2 (PRC2) and is shared by the methylation signatures of DNA and histone marks [27] emerges as a possible druggable target.

## RESULTS

Our working hypothesis was that the phenotypical reversion of EMT (MErT) characterises the epithelial variant of peritoneal mesothelioma (E-MpM), and that this variant is plastic and has some of the traits of CSCs. To address this hypothesis, we analysed surgical specimens (17 E-MpMs, 2 HG E-MPM and 2 S-MpMs, Table 1) obtained from 21 untreated patients, using an integrated approach including immunohistochemistry (IHC), immunofluorescence, in situ hybridization (ISH), biochemistry and human pluripotent cell array. Cultured E-MpM tumour cells derived from the specimens were then used to confirm *in vivo* observation on stemness and plasticity by challenging them with cytokines and growth factors as well as small drugs (bevacizumab, sunitinib and GSK 126).

**Table 1:** IHC results

		VEGFR2 exp/act	MET exp/act	c-MYC	SLUG	TWIST	EZH2	E-Cad
**1**	Omentum	+/na	+/na	+	−	−	−	**+/−**
**2**	Omentum	+/na	+/na	+	−	−	−	**+/−**
**3**	Omentum	+/na	+/na	+	−	−	−	**+/−**
**#1**	E-MPM 1^Δ^	+/−	+/+	+	−	−	+	+
**#2**	E-MPM 2^Δ^	+/−	+/+	+	−	−	+	+
**#3**	E-MPM 3^Δ^	+/−	+/+	+	−	−	+	+
**#4**	E-MPM 4	+	+	+	−	−	+	+
**#5**	E-MPM 5	+	+	+	−	−	+	+
**#6**	E-MPM 6^Δ^	+	+	+	−	−	+	+
**#7**	E-MPM 7	+	+	+	−	−	+	+
**#8**	E-MPM 8	+	+	+	−	−	+	+
**#9**	E-MPM 9	+	+	+	−	−	+	+
**#10**	E-MPM 10	+	+	+	−	−	+	+
**#11**	E-MPM 11^Δ^*	+/−	+	+	−	−	+	+
**#12**	E-MPM 12	+	+	+	−	−	+	+
**#13**	E-MPM 13^Δ^*	+/−	+	+	−	−	+	+
**#14**	E-MPM 14^Δ^	+	+/+	+	−	−	+	+
**#15**	E-MPM 15^Δ^	+/+	+/+	+	−	−	+	+
**#16**	E-MPM 16^Δ^* progressed	+	+	++	−	−	++	+/−
**#17**	E-MPM 17^Δ^ progressed	+	+	++	−	−	++	+/−
**#18**	HG E-MPM 1	−	−	++	+f	+	+++	+f
**#19**	HG E-MPM 2	−	+	+++	−	−	+++	−
**#20**	S-MPM 3^Δ^	−/−	−/−	−	+++	+++	++	−
**#21**	S-MPM4 ^Δ^	−/−	−/−	−	+++	+++	+	−

exp/act: expression/activation;

+/na: activation not achievable in fresh omentum samples;

+/−: expressed but not activated;

+/+: expressed and activated;

f: focal expression

Δ VEGFR2 and Met activation was investigated in snap-frozen fresh surgical samples;

* Fresh samples used for primary cell cultures

Cases 16 and 17 were defined as progressed because they showed a greater degree of malignancy than E-MpM cases #1-15, but were less malignant than the high-grade (HG) E-MpM cases #18 and #19.

### Surgical specimen analyses

#### Comparison of mesothelium and E-MpM expression profiles

**Figure 1:**
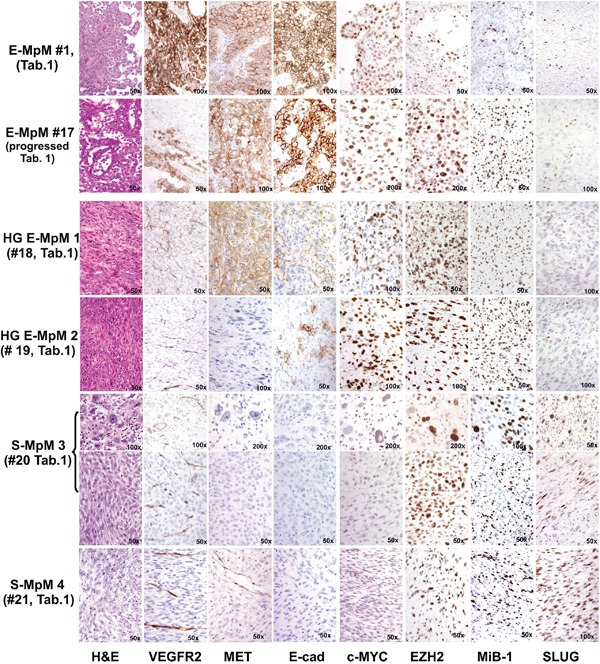
IHC findings The figure compares a classic E-MpM (#1, Table 1) and one progressed E-MpM case (#17, Table 1) with two HG E-MpMs (#18 and #19, Table 1) and two S-MpMs (#20 and #21, Table 1). The classic E-MpM is characterised by a positive triple immunophenotype including VEGFR2, c-MET and E-cadherin, whose intensity and the number of immunolabelled cells decreases in the progressed case, become even less in the HG E-MpM, and disappear in the S-MpM. The reverse is true in the case of c-MYC, EZH2 and Mib-1, which decorate an increasing number of cells going from E-MpM to HG E-MpM. SLUG is constantly negative. On the other hand, S-MpM always has a negative triple immunophenotype and c-MYC immunostaining, but show SLUG positivity, and the expression of EZH2 and Mib-1 are similar to that in HG E-MpM. S-MpM3 has one pleomorphic and one spindle area (identified by parentheses). The image gallery of three additional cases of E-MpM (#11, #13 and #16) shown in Supplementary Figures 4, 5 and 7 shows that VEGFR2 /c-MET and c-MYC/EZH2 immunoprofiles are superimposable in the surgical specimens and their corresponding cell lines. Original magnifications: 50X, 100X and 200X (as indicated in each figure).

Immunohistochemical analysis revealed that VEGFR2 and c-MET were expressed by the E-MpM specimens and mesothelium (Table 1 and Figure 1), in line with recent reports [12, 13], and the same was true of the pleiotropic marker c-MYC [22, 23, 28]. In the mesothelium, E-cadherin expression was faint and heterogeneous, whereas the EZH2 catalytic unit of PRC2 [26], which has been reported to be expressed at low levels in normal cells [26, 29], was undetectable and Slug was negative (Supplementary Figure S1). With the exception of the undetectable EZH2, non-activated mesothelium seems to bear the basic machinery exploited by E-MpMs.

#### Immunoprofiles of the surgical specimens of MpM variants

EZH2 was expressed by the entire spectrum of MpM surgical specimens, although to different extents (Table 1 and Figure 1). The loss of INI1-mediated EZH2 upregulation [30] was excluded by IHC (not shown). Unlike that of EZH2, the expression of OMSK cocktail-related c-MYC transcription factor (TF) [22], the receptor tyrosine kinases (RTKs) c-MET and VEGFR2, and the adhesion molecule E-cadherin segregated with the E-MpM variant (Table 1 and Figure 1).

In terms of cell immunostaining distribution, c-MET expression was mainly restricted to cell membranes, whereas the expression of VEGFR2 was both membranous and cytoplasmic, thus suggesting that it may play a non-canonical role. The expression of both markers was confirmed by ISH at RNA level (Supplementary Figure S2). E-cadherin showed membrane reactivity, in line with its role in promoting pluripotency by means of cell-to-cell adhesion [19, 21, 31]. The percentage of nuclei immunolabelled by the specific c-MYC and EZH2 antibodies was similar in most of the E-MpM specimens, with an increase paralleling that of Ki-67 restricted to cases #16 and #17 (Table 1, Figure 1). This finding suggests that these two cases may represent an intermediate point in the spectrum of progression toward the HG/undifferentiated form of E-MpM (see below).

The S-MpM specimens (cases #20 and 21, Table 1) showed the loss of E-cadherin as well as c-MYC, VEGFR2 and c-MET and were enriched in cells expressing the SLUG (Figure 1) and TWIST (not shown) nuclear TFs, with a large percentage of nuclei decorated by EZH2 and Ki-67 (Figure 1).

The immunophenotypical analysis highlighted the fact that not all E-MpMs progress to S-MpMs, but there is a type of upgraded form of E-MpMs that retains a immunoprofile more close to E-MpM (cases #18 and #19, Table 1, Figure 1). The increase in EZH2 in HG MpMs is consistent with the reported increase in the level of TGFbeta (see below and Figure 5), its acknowledged role in maintaining CSCs [32] and the decreased expression of E-cadherin [33]. Taken together, these findings suggest that c-MYC or EMT regulators respectively concur in MErT and EMT via the EZH2 chromatin modifier. The c-MYC findings are in line with its role as a core pluripotency factor [22], its ability to interact with EZH2 [23] and to facilitate an epithelial phenotype and MErT (at least in mouse) [34].

#### miRNA 17-5p in situ hybridisation

**Figure 2:**
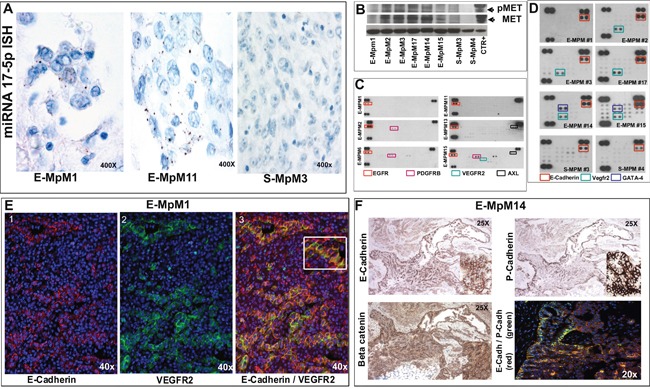
ISH, biochemical and Immunofluorescence findings **A.** The positive miRNA-17-5p ISH signal seen as brown dots localised in the cytoplasm of two E-MpMs (#1 and #11) is absent in the S-MpM (#3). **B.** MET WB analyses of the frozen material of the E- and S-MpMs (CTR+: A431 cell line) indicated in Table 1. **C.** Notice the absence or very low activation of VEGFR2 (as demonstrated by the Proteome Profiler™ Array readouts). **D.** The human pluripotent stem cell array confirmed the presence of E-cadherin and VEGFR2 (cases #1, #2, #3 and #17) and revealed GATA-4 expression in E-MPMs #14 and #15. Unlike IHC, the stem cell array showed that the E- and S-MpMs (#3 and #4) expressed E-cadherin. This discrepancy may be attributable to a difference in the E-cadherin epitopes recognised by the IHC antibody (clone NCH-38, M3612, Dako) and the R&D E-cadherin antibody present in the array but, as both antibodies are patent protected, no information is available regarding the epitopes used as immunogens. **E.** E-cadherin (red) and VEGFR2 (green) immunofluorescence highlights the hybrid phenotype (image 3 and inset) and the heterogeneity of E-MpM (images 1, 2 and 3). **F.** E- and P-cadherin co-expression and beta catenin membraneous decoration in serial sections of E-MpM #14. See text for further details. Original magnifications: 20X, 25X, 40X and 400X (as indicated in each figure).

As the observed modulation of c-MYC in E-MPMs and S-MPMs paralleled the level of miRNA 17-5p, we used ISH to investigate miRNA 17-5p expression in two cases of E-MpM (#1 and #11, Table 1) and one of S-MpM (S-MpM3, case #20, Table 1). This miRNA belongs to a cluster of miRNAs in human chromosome 13 that has been reported to be activated by c-MYC [35]. The two E-MpMs expressed high levels of miRNA 17-5p, with granular cytoplasmic expression in less than 25% of the tumoral cells and signal intensity ranging from moderate to strong (score ≥6), whereas no signal was expressed by the S-MpM (Figure 2A). These findings complement previous observations [35] and reinforce the idea that miRNA 17-5p in E-MpMs has oncogenic activity supporting the capacity of miRNAs to influence tumorigenesis in a cell-specific manner [36].

#### Biochemistry

Using the cryopreserved material obtained from each halved surgical sample before fixation, we used Western blotting (WB) to confirm MET and VEGFR2 expression, and interrogate the activation status of c-MET (Figure 2B) and VEGFR2. Unexpectedly, very little or no activation of VEGFR2 was observed (Figure 2C). To address this issue, we first investigated VEGFR2/MET co-immunoprecipitation in order to explore the hypothesis of VEGFR2 silencing- induced phosphatase-mediated MET up-regulation [37]. As both analyses were unsuccessful (data not shown), we made *in vitro* analyses of cells obtained from frozen surgical material (see Figure 4, VEGFR2 switching off) in order investigate possible stemness in E-MpMs. Recent studies have shown that sunitinib-mediated VEGFR2 silencing participates in regulating pluripotency in embryonic stem cells [38] and triple-negative breast cancer [39], and that a stable mesothelioma cell line derived from a patient over-expressing VEGFR2 and c-MYC had stem-like traits [15].

#### Human pluripotent stem cell array

Pluripotent stem cell array analysis of the frozen material also used for the WB experiments revealed three factors in a ground state and therefore characteristics of CSCs: VEGFR2, which was expressed in E-MpM; E-cadherin, a MErT/EMT regulator that was shared by E-MpMs and S-MpMs despite its irrelevant immunophenotypical expression in S-MpMs; and GATA4, a lineage control transcription factor that activates or represses depending on the cell context [40, 41] and was restricted to two E-MpM cases (#14 and #15, Table 1, Figure 2D).

Together with the immunophenotype data, the stem cell array readouts pointed at E-cadherin as a stemness regulator (attributable to its ability to form cell-cell adhesion exchanges) [19], and support a pluripotency-related role of dephosphorylated VEGFR2.

#### Hybrid immunophenotyping

In the light of the recent suggestion that the hybrid epithelial/mesenchymal (E/M) phenotype plays a key role in stemness as well as in MErT and EMT [6], we applied IHC and immunoflourescence to investigate the distribution of E-cadherin and VEGFR2 in individual E-MpM tumoral cells of the histological fixed samples.

The IHC and immunofluorescence analyses showed that, although some cells were decorated by E-cadherin or VEGFR2 alone, a considerable number co-expressed both markers (case #1, Table 1, Figure 2E and Supplementary Figure S3). This confirms the hybrid phenotype, highlights E-MpM heterogeneity, and suggests that the tumour may endow the cells with some of the characteristics of stemness and plasticity.

Furthermore, as it has been shown that P-cadherin expression is crucial to identifying the intermediate EMT state and cells with CSC properties when concomitantly expressed with E-cadherin [42], we investigated our samples using P-/E-cadherin antibodies and broadened the panel to include beta-catenin. P-/E-cadherin immunodecoration and immunofluorescence co-labelling proved to be restricted to E-MpMs and, once again, had a patchwork distribution and beta-catenin immunostaining revealed membrane expression, as previously reported [24] (Figure 2F).

Taken together, these findings confirm that the EMT in cancer has features of plastic process with multiple intermediate states rather than a “all or nothing” response [6], and support the idea that E-MpM is governed by its conversion to MErT.

**Figure 3:**
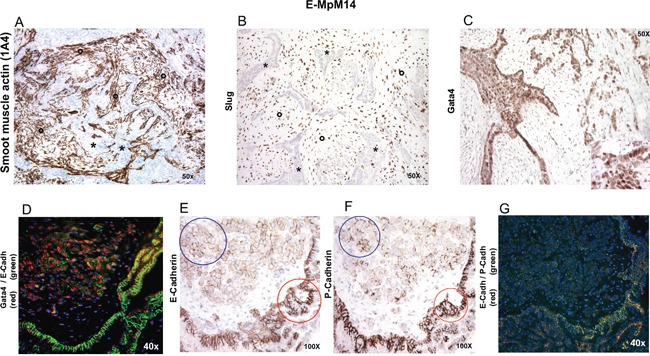
IHC and IF characterisation of stromal and tumoral cells in one GATA-4-positive case E-MpM #14 (Table 1) showed a well-developed stromal component enriched in cancer-associated fibroblasts (indicated by asterisks) expressing smooth muscle actin **A.** and SLUG **B.** Note that the stroma-embedded, epithelioid-like tumoral component (indicated by circles) showed strong GATA-4 nuclear immunoreactivity **C** and **D.** and lower E-cadherin (blue circle, **E.** and P-cadherin (blue circle, **F.** expression than the surrounding papillary/row featuring epitheliod tumoral cells (red circles, 3E and 3F). The results were confirmed by immunofluorescence **G.** Similar results were obtained in three other E-MpMs (#6, #7 and #15, Table 1) that showed GATA-4 expression. Original magnifications: 40X, 50X and 100X (as indicated in each figure).

Interestingly, the two cases expressing GATA4 as revealed by the pluripotent stem cell array (#14 and #15, Table 1) had a well-developed stromal component that was enriched in cancer-associated fibroblasts (CAFs) expressing smooth muscle actin (SMA) [43] and characterised by SLUG decoration. They also had a tumoral stromal component in which single cells expressed lower levels of E-cadherin and P-cadherin, and showed nuclear GATA4 immunoreactivity, findings that were confirmed by the co-immunofluorescence analysis (Figure 3). The same immunostaining pattern was seen in two other cases (#6 and #7, Table 1) with a well-developed, multifocal stromal component revealed by hematoxylin and eosin. It is worth noting that FISH analysis showed that all four cases had a diploid profile in the CAF component (not shown), unlike the tumoral cells that presented at least one FISH abnormality (Supplementary Table S1).

### Cell cultures

In order to investigate stemness and plasticity further, we used short-term cultures and stable cell lines obtained from the same surgical specimens as those analysed by means of IHC, immunofluorescence, biochemistry and the pluripotent stem cell array.

#### Characterisation of two short-term cell lines and one spontaneous stabilised cell line

Starting from fresh E-MpM surgical samples, two short-term cell lines (STC1 and STC2, corresponding to E-MpM cases #11 and #13, Table 1) and one spontaneous stable cell line (SSL1, corresponding to case #16 E-MpM) were established, characterised, and used in *in vitro* experiments. STC1 (Supplementary Figure S4) and STC2 (Supplementary Figure S5) both consisted of large, round, epithelial-like cells, and had an immunophenotype that was fully consistent with E-MpM, except for the activation of VEGFR2 shared by both cell lines and SLUG expression restricted to STC1 (Supplementary Figure S6). The spontaneously obtained SSL1 cell line was mainly made up of spindle-shaped cells but also included cells with epithelioid features (Supplementary Figure S6). Its immunophenotype was consistent with E-MpM in terms of c-MET, c-MYC and EZH2 expression, but E-cadherin levels were low, SLUG was expressed, and there was no VEGFR2 expression (Supplementary Figure S6-S7). This phenotype, which strongly suggested an attenuated epithelioid phenotype consistent with MErT, and a transition towards an intermediate rather than a full EMT phenotype, was not unexpected as the cell line was derived from case #16, the most “progressed” of the E-MpMs.

As this intermediate (epithelioid/mesenchymal) phenotype was shown by the cells immediately after seeding and was maintained during the passages (in terms of cytokeratin CAM 5.2/CD44 co-expression, data not shown), we concluded that SSL1 is a stable hybrid cell population co-expressing epithelial (CAM 5.2)/mesenchymal as well as CSCs (CD44) markers. It is worth noting that compared to the surgical specimens from which they were derived the cell lines had a more mixed profile including features of both HG E-MpM and S-MpM. These changes probably represent an incomplete switch towards a mesenchymal phenotype endowed with high degree of plasticity because of the partial EMT [6].

#### Stemness and plasticity experiments

Given the characteristics of the short-term cultures and the stable cell line, we used the first (which showed activated VEGFR2) to investigate whether phosphorylation had an impact on stemness, and the second (which had more mesenchymal traits) to verify the more metastable plasticity of E-MpMs.

#### The pharmacological switching off of VEGFR2

**Figure 4:**
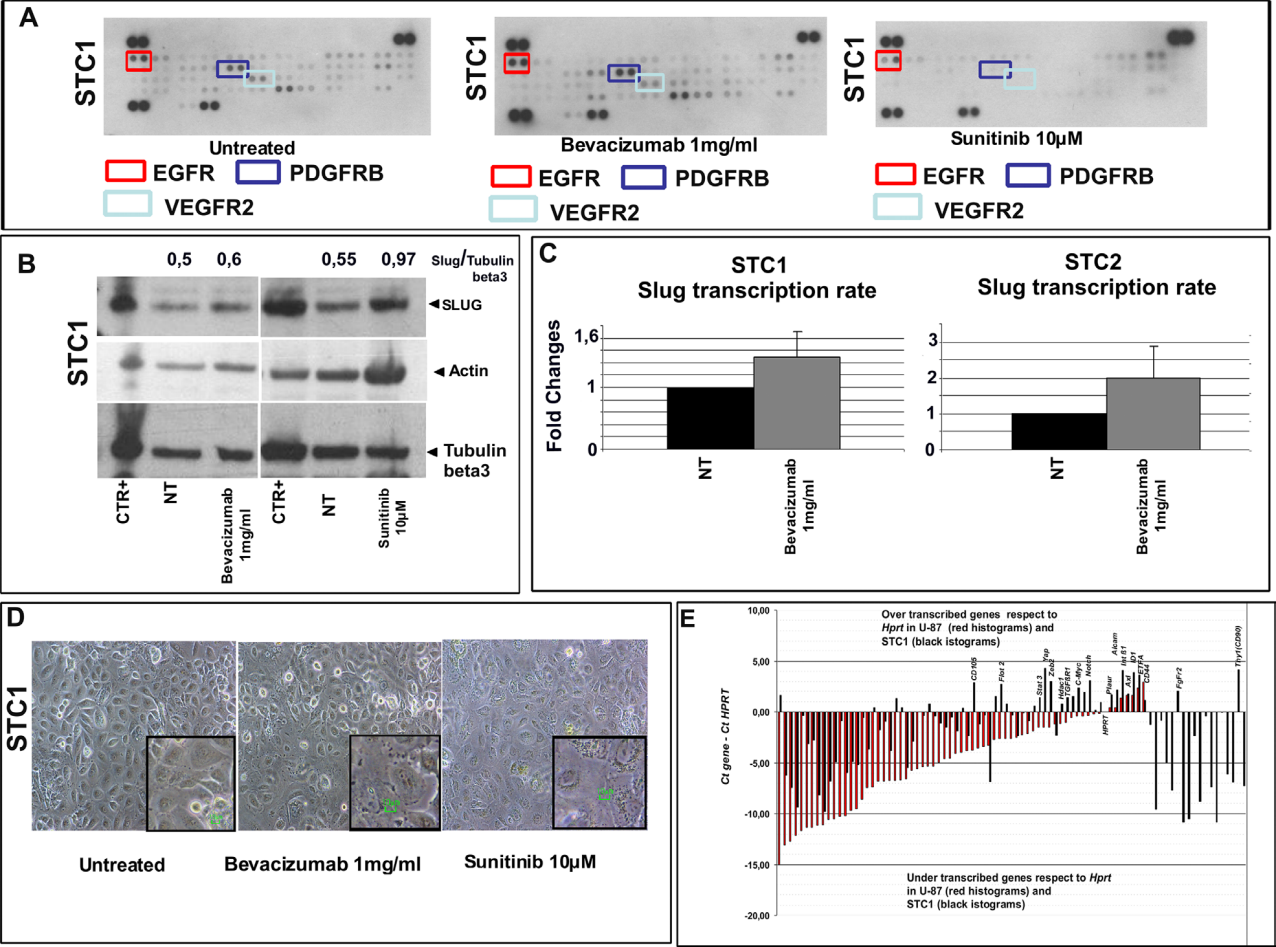
Switching off VEGFR2 regulates the stemness traits of E-MpMs **A.** STC1 was treated with bevacizumab and sunitinib for 24 hours. The relatives changes in VEGFR2 and PDGFRB activation were calculated using *ImageJ* software by normalising the corresponding dots in treated and untreated samples. After bevacizumab treatment, PDGFRB and VEGFR2 respectively showed activation (from 8.25 to 9.26) and deactivation (from 6.92 to 6.40). After sunitinib treatment, both PDGFRB and VEGFR2 were completely de-phosphorylated. Both drugs induced an increase in SLUG (the relative changes in SLUG expression were calculated using *ImageJ* software with tubulin beta3 as a housekeeping protein (shown in the figure as normalised SLUG/tubulin beta3). **B.** As the increase of SLUG transcription measured in STC1 by quantitative PCR is small, it can be assumed that it is unable to induce a complete EMT **C.** an assumption that is supported by the similarly treated STC2 (4C) and the fact that STC1 retained its epithelioid morphology **D. E.** The transcriptional level of various genes known to be involved in stemness was investigated by comparing STC1 with the U-87 glioma cell line (a tumour known to have a strong CSC component). The complete gene list is given in RT^2^ Profiler PCR array cancer stem cells (Qiagen, Cat. No. PAHS-176Z). *Hprt* was chosen as the reference house-keeping gene because its transcription level was similar in STC1 and U-87 (i.e. a cycle threshold of about 24 in both lines). The most significantly over-transcribed genes are shown in the figure. The results underline the transcriptional enrichment of mesenchymal (*Thy1, FGFR2, AXL, ALCAM* and *CD105*) and staminal genes (*c-MYC and ID1*) in STC1 in comparison with U-87. Original magnification 10x; scale bar 10 μM (inset).

**Figure 5:**
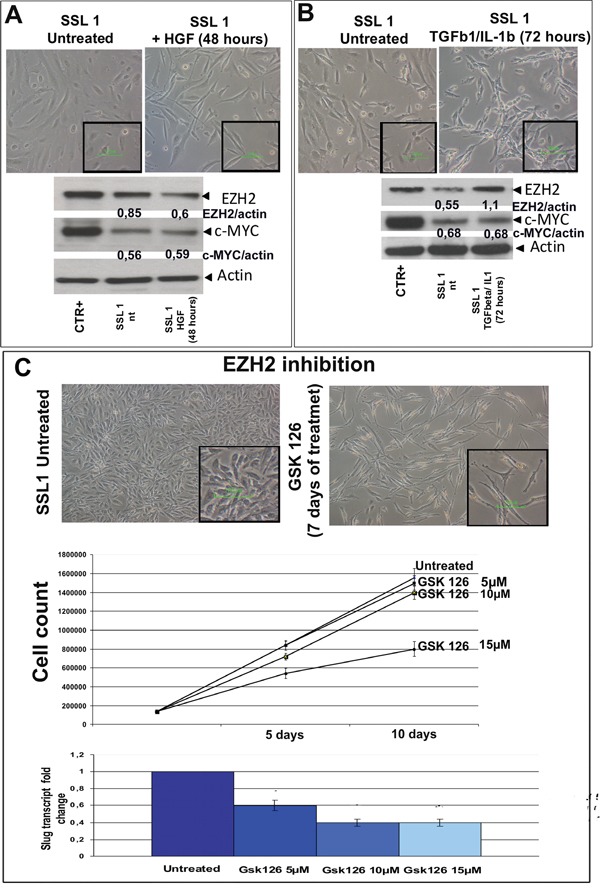
HGF, TGFβ1/IL1b and GSK 126 treatment **A.** SSL1 was starved by means of 48-hour serum restriction, and then treated with recombinant HGF (20 ng/mL) for 30 minutes. Despite the minimal morphological changes, the WB readouts showed a decrease in EZH2 while c-MYC remained stable. Original magnification 20x; scale bar 100 μM (inset). **B.** SSL1 was starved by means of 48-hour serum restriction, and then treated with recombinant TGFβ1 (0.5 ng/mL) and IL1b (2 ng/mL). After treatment, the cells acquired more spindle-shaped features, and WB revealed a marked increase in EZH2. c-MYC remained stable as after HGF treatment. Original magnification 20x; scale bar 100 μM (inset). The relative changes in EZH2 and c-MYC expression were calculated using *ImageJ* software with actin as a housekeeping protein (shown in the figure as normalised EZH2/actin or c-MYC/actin). **C.** SSL1 was treated with GSK 126 as shown in the figure. After seven days of treatment, the cells acquired a more elongated and bi-polar morphology. After 10 days of treatment, a reduction in cell growth and SLUG transcription were respectively observed by means of cell counts and quantitative PCR. Original magnification 20x; scale bar 10 μM (inset).

STC1 and STC2 were treated for 24 hours with bevacizumab (a specific VEGFR2 inhibitor) and sunitinib (an inhibitor of the PDGFR family, VEGFR2 and c-MET). In addition to reduce the activation of their targets (stronger in sunitinib respect to bevacizumab treatments, Figure 4A), both drugs induced an increase in SLUG (Figure 4B). Overall, the experiments demonstrated that VEGFR2 activation induces SLUG degradation, and that its switching off leads to a more primitive CSC-like status as shown by the increase in SLUG protein and RNA (Figures. 4B and 4C), even if this did not result in full EMT. Further support to the concept of incomplete EMT was given by the retained epithelioid morphology of STC1 and STC2 after VEGFR2 had been switched off (compare Figure 4D with Figure 5).

#### CSC array-based comparative analysis

As pluripotent genes are critical for maintaining pluripotency, we used CSC array to compare the number of CSC genes between an E-MpM and glioblastoma cell line (which is known to harbour a high percentage of CSCs) [44] as a means of further confirming that E-MpMs are enriched in CSC cells. The results showed the transcriptional enrichment of genes known to be over-expressed in mesenchymal stem cells (*Thy1, Fgfr2, Axl, Alcam* and *CD105*) and genes involved in controlling pluripotency (*c-MYC* and *ID1*) in STC1 (Figure 4E).

#### MpM plasticity

After HGF stimulation, SSL1 showed a decrease of EZH2 expression (Figure 5A). Conversely, the treatment of SSL1 with TGFbeta1/IL1 induced an increase in EZH2 (Figure 5B). On the contrary, c-MYC levels remained stable during both treatments.

Overall, these findings are in keeping with the idea that MErT and EMT are transient and reversible conditions, and that mesothelioma cells can feature a dynamic phenotype that allow them to shift from more epithelioid (MErT) or more mesenchymal traits (EMT) and back.

#### EZH2 pharmacological inhibition by GSK 126

To verify whether EZH2 inhibition could be therapeutically exploited in E-MpM, we treated SSL1 (the spontaneous stabilised cell line obtained from an epitheliod mesothelioma) with the EZH2 inhibitor GSK 126, which lead to a reduction in growth and a decrease in SLUG transcription (Figure 5C).

## DISCUSSION

Most previous studies have focused on the role of EMT and very few have investigated MErT, particularly in the context of mesothelioma. Our findings provide evidence that E-MpM is enriched in stemness and plasticity governed by an MErT process.

MpM arises from the mesothelium, a mesenchymal tissue derived from the celomic epithelium expressing WT1 [45] that has an intrinsic ability to differentiate along both the epithelial and mesenchymal axes. This trait is preserved in MpM. We began our analysis by investigating the status of c-MET, which is known to favour mesenchymal-epithelial transition [17], in a series of surgical specimens. The expression/activation of c-MET proved to be restricted to E-MpM and, focally and to a very little extent, its direct high-grade (HG) progression. The HGF/c-MET activation loop, that possibly reflects the cell response to asbestos-induced DNA damage, triggers mesothelial cell reprogramming. This phenomenon may directly affects bivalent mesothelial cells [45] but also mesothelial cells that have acquired a myofibroblast phenotype after a first EMT round triggered by an inflammatory asbestos-induced repair response [46].

The aim of the subsequent surgical specimen-based analyses was to identify the interactions between chromatin-modifying factors, TFs, adhesion molecules and RTKs, and showed that EZH2 was increasingly expressed from E-MpM to HG E-MpM and S-MpM, whereas c-MYC, E-cadherin and VEGFR2 expression was restricted to E-MpMs. The expression of c-MYC not only increased within the E-MpM spectrum, but also closely correlated the expression of miRNA 17-5p, a non coding c-MYC regulated RNA that has been reported to contribute to highly malignant tumours in humans and promote stem cell properties in mice [37]. On the basis of these findings, and bearing in mind the spectrum-wide role played by c-MYC [15, 22, 23, 28, 36] and the typical wide spread presentation of MpM, we hypothesised that c-MYC may facilitate and maintain [47] the CSC-like status of E-MpM cells during their characteristic metastatic-like seeding of the abdominal cavity [11] that acts as hospitable soil similar to an enlarged niche [48].

Further robust support of the stemness of E-MpM was given by the pluripotent stem cell array readouts, which allowed us to investigate the existence of the hybrid E-cadherin/VEGFR2 phenotype that may provide E-MpM cells with stemness and plasticity by exploiting epithelial-related cell-cell adhesion and mesenchymal-related migration. The membranous decoration of E-cadherin, P-cadherin [6, 42], and beta-catenin [24] also supported the presence of the E/M phenotype and its association with stemness and plasticity (Figure 3).

The results also suggested that the progression to HG E-MpM may occur in two different ways. In the first case, which may correspond to that described as transitional or, more probably HG/undifferentiated MpM [11], the E-MpM cells retain the immunophenotypical profile but activate c-MYC and EZH2. This correlates with a high tumor proliferation index (Ki-67) and, due to EZH2 overexpression, a decrease in E-cadherin (Table 1, cases #18 and #19) [49]. The hypothesis of direct progression is supported by evidence of E-MpM cases with increasingly malignant characteristics, such as our cases #16 and #17. The second type of progression is the progression to S-MpM. In this case, c-MYC is replaced by the expression of the EMT regulators SLUG and TWIST, with down regulation of E-cadherin, VEGFR2 and c-MET. The modulation of the immunoprofile is in line with the ability of c-MYC to counteract the EMT pathway [50] and, as happens in embryonic stem cells [51], interfere with the epigenetic regulation of CSCs [52].

**Figure 6:**
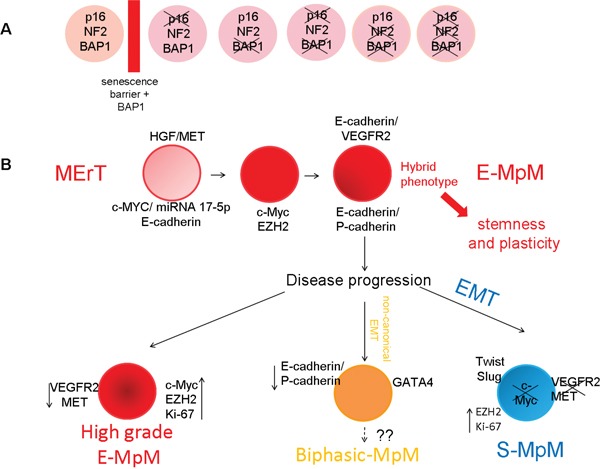
Proposed E-MpM model The model suggests that, once having overcome the senescence barrier **A.**, (see also Supplementary Table S1) which *per se* significantly increases the yield of CSC colonies, the cells enter MErT to up-regulate pluripotency as a result of a c-MET/HGF loop **B.** The expression of c-MYC, miRNA 17-5p and E-cadherin seems to be necessary and sufficient to trigger the initial steps of MErT. Thereafter, the interaction of c-MYC and EZH2 (acting as surrogate epigenomic mediators determining cell fate) allows and maintains the hybrid E-cadherin/VEGFR2 phenotype (the hallmark of E-MpM) and the concomitant expression of P- and E-cadherin. As demonstrated by our cell culture experiments, these hybrids are endowed with stemness traits and a high degree of plasticity. During disease progression, in keeping with the plasticity of MErT/EMT processes, the hybrid MErT phenotype can progress to high-grade E-MpM or acquire the full EMT leading to S-MpM. This parallels the decommissioning c-MYC, which is replaced by EMT regulator expression (SLUG and TWIST). As a lineage control factor, GATA-4 may be involved in resetting progression, and be a prerequisite for biphasic E-MpM (E-MpM showing separated areas of both sarcomatous and epithelial elements).

Overall, our findings suggest a model in which the reprogramming is driven by MErT in E-MpM and by EMT in S-MpM (Figure 6). During such reprogramming, it is thought that the interaction between TFs and the epigenetic regulators is essential to induce the decommission of their poised status in order to activate/repress transcriptionally the specific context-dictated epithelial/mesenchymal genes. Here, the c-MYC and EMT regulators representing TFs and the histone protein EZH2 acting as a surrogate of epigenetic regulators, while P-/E-cadherin and VEGFR2 cooperate in giving a hybrid phenotype that endows E-MpM cells with stem cell characteristics.

In addition to E-/P-cadherin and VEGFR2, two our E-MpMs investigated by means of pluripotent stem cell array also showed the expression of GATA4, another stemess-related marker. The signature of these two cases (subsequently joined the two fixed cases #6 and #7, Table 1) closely mirrored that of E-MpM but they also had a well-developed stromal component enriched in CAFs expressing SLUG and TWIST, thus supporting the idea that CAFs may elicit the acquisition of stem-like traits and/or favour EMT [53, 54]. No evidence of malignancy of the stromal component was found in any of these cases, but the tumoral cells showed nuclear immunoreactivity when challenged with GATA 4 antibody and retained, albeit decreased, E-cadherin decoration. The interplay between GATA4 and E-cadherin is in line with the non-canonical GATA4-type of EMT in which GATA4 triggers a E-cadherin-mediated redistribution that affects adhesion and facilitates collective cell migration without interfering with the RNA or protein levels of E-cadherin [55]. These findings further support the hybrid nature of tumoral cells and raise the hypothesis that the observed changes may represent an early phase of progression to a biphasic variant of MpM.

Short-term cultures allowed us to identify a possible unprecedented role for VEGFR2. Whilst canonical VEGFR2 activation is typically marked by SLUG degradation, VEGFR2 novel and activation-independent activity is associated with SLUG expression (Figure 4). It is worth noting that the extent expression of SLUG was moderate, and this is in line with the partial, hybrid phenotype known to endow tumour cells with stemness [6]. These results disregard the expectancies generated by the recently re-proposed vascular featuring immunophenotype of E-MpM (VEGFR2 expression and/or VEGFR2/MET co-expression in E-MpM) [12] for a role of anti-angiogenic treatments.

The hypothesis that E-MpM is made up of cells with stem-like properties was also supported by the results obtained by applying the cancer stem cell profiler complemented by PCR to STC1. The readouts highlighted the transcriptional enrichment of genes known to be involved in maintaining pluripotency (*c-MYC and ID1*) [22, 56] or highly expressed in mesenchymal stem cells (*Thy1, Fgfr2, Axl, Alcam* and *CD105*) [57–59]. Furthermore, our SSL1 experiments demonstrated that E-MpM has a high degree of plasticity: when challenged with TGFβ1/IL1b, the tumoral cells acquired a more mesenchymal phenotype but, when challenged with HFG, they acquired a more epithelioid phenotype. Finally, given the biological characteristics of our E-MpM specimens, we treated the most spindle cell-enriched E-MpM-derived SSL1 cell line with the EZH2 inhibitor GSK 126. The observed reduction in cell proliferation strongly suggests that it is worth considering the possibility of using chromatin modifiers or inhibitors to treat patients with mesotheliomas.

On the basis of our findings as a whole, we propose a model in which E-MpM may be viewed as a disease essentially composed by cells with an hybrid phenotype within the MErT framework. In this model, rather than corresponding to a more differentiation-committed entity, E-MpM represents a state of greater, plastic, stemness-enriched tumoral growth that is likely to be poorly responsive to traditional treatment but may respond to epigenetic regulator inhibitors. This possibility is further supported by the recently reported inverse correlation between strong EZH2 expression and the loss of the chromatin modifier BAP-1 [60], whose increasingly deregulated nature in sporadic mesotheliomas [61] we are currently investigating.

## MATERIALS AND METHODS

### Patient samples

The case material consisted of formalin-fixed, paraffin-embedded (FFPE) surgical samples obtained from 21 previously untreated patients (who subsequently underwent cytoreductive surgery and hyperthermic intraperitoneal chemotherapy).

On the basis of the proposed TMN staging of MpM [62], all but one patient (case #12, Table 1) were in stage 2 (10 patients) or 3 (10 patients). The diagnoses made on the basis of morphological and immunophenotypical criteria (calretinin, WT1, cytokeratin 5/6) [11] as well as podoplanin as proposed in the WHO classification [10] were 17 E-MpMs (#1-17, Table 1), two HG E-MPM (#18-19, Table 1) and two S-MpMs (#20-21, Table 1). Frozen material was available for biochemical analyses of 11 patients, three of whom also provided additional fresh material for the primary cell culture experiments. Finally, three FFPE omentum samples were obtained from unrelated MpM patients. Written informed consent was obtained from all of the patients in accordance with the regulations of the Ethics Committee of the Fondazione IRCCS Istituto Nazionale dei Tumori, Milan, Italy.

### Immunohistochemistry (IHC), fluorescence *in situ* hybridisation (FISH) and PCR assessments of p16, NF2 and BAP1

All of the cases were analysed for *P16* and *NF2* gene status by FISH, *P16* gene promoter methylation by means of PCR, and p16 and BAP1 expression by means of IHC. The methodological details and results are given in the supplementary material and Supplementary Tables S1 and S2. In brief, 18 cases had overcome senescence: 12 cases showed the inactivation of *P16* (detected by means of FISH and/or promoter methylation and/or IHC), 6 cases the loss of *BAP1* and 1 case the loss of *BAP1/NF2*. The remaining three cases showed the loss of *BAP1*, together with the loss of *NF2* in one case (Supplementary Table S1).

#### Immunohistochemistry

Representative 2 μm sections obtained from FFPE tumoral samples of all of the cases were selected and phenotyped. The antibodies and experimental conditions used to detect the expression of VEGFR2, c-MET, E-cadherin, c-MYC, EZH2, Ki67, SLUG, TWIST, P-cadherin, beta catenin, 1A4 actin, GATA4, p16, BAP1 and INI1 are shown in Supplementary Table S2.

#### In situ hybridization (ISH)

The c-MET RNA, VEGFR2 RNA and microRNA (miRNA) 17-5p ISH methods are described in the supplementary material.

#### Immunofluorescence

The co-expression of E-cadherin, VEGFR2, E-cadherin/P-cadherin and E-cadherin/GATA4 was investigated by means of immunofluorescence. The primary antibodies were diluted and retrieved as described in the supplementary material, and the slides were then incubated for one hour at room temperature with the specific secondary Alexa Fluor antibody (Alexa Fluor 488 and 546, Thermo Fischer Scientific, MA, USA).

#### Western blotting and phospho-Receptor tyrosine kinases (RTKs) array

All of the analyses were made using frozen material taken from the patient samples (Table 1). VEGFR2 activation was investigated using a phospho-RTK array kit (Proteome Profiler™ Array, ARY001B, R&D Systems, Minneapolis, MN, USA) and 1 mg of protein lysate in accordance with the manufacturer's protocol. The Western blot (WB) conditions used to detect c-MET, c-MYC, EZH2, SLUG and β-actin expression and c-MET activation are shown in Supplementary Table S3.

Protein amounts were evaluated using ImageJ software in accordance the manufacturer's instructions.

#### Human pluripotent stem cell array

Stem cell marker expression was assessed by means of a human pluripotent stem cell array kit (Cat. No. ARY010 R&D Systems) in accordance with the manufacturer's instructions.

### Primary cell cultures

#### Establishment and characterisation

Two short-term cell cultures and one stable cell line were obtained starting from the fresh material of three patients (Table 1) and characterised by means of WB, flow cytometry and IHC as described in the supplementary material.

#### RNA analyses

RNA extracted from mesothelioma cell cultures and the U-87 glioma cell line (American Tissues Cell Cultures, Cat. No HTB-14), was quantified and retro-transcribed as described in the supplementary material. The cancer stem cell genes were investigated using RT^2^ Profiler PCR array cancer stem cells (Cat. No. PAHS-176Z, Qiagen, Hilden, Germany) in accordance with the manufacturer's guidelines. Selected genes were also investigated by means of real-time PCR as described in the supplementary material.

#### Bevacizumab and sunitinib treatments

Bevacizumab (Avastin, Roche, Basel, Switzerland) 25 mg/mL was diluted to 1 mg/mL in cell culture medium; sunitinib (Cat. No. S1042, Sellek, Munich, Germany) was diluted to 10 mM in DMSO, and 10 μM used for treatment. After 24 hours of treatment, the proteins were extracted as described in Supplementary Table S2, and 20 μg of the cell extract was investigated by means of WB using the SLUG, actin and Tubulin beta3 antibodies (Supplementary Table S3). VEGFR2 and PDGFRB activation was investigated using a phospho-RTK array kit (Proteome Profiler™ Array, ARY001B, R&D Systems).

#### Hepatocyte growth factor (HGF) treatment

Before treatment, the cells were deprived of serum for two days. Recombinant HGF (Cat. No. H1404, Sigma-Aldrich, Saint Louis MO, USA) was added in cell culture medium at a concentration of 20 ng/mL [63] for 30 minutes, and then removed. The proteins were extracted from untreated or HGF-stimulated cells after 48 hours, and investigated by means of WB as described in Supplementary Table S3.

#### Transforming growth factor beta1 (TGF-b1) / interleukin 1b (IL-1b) treatment

After being deprived of serum for two days, the cells were treated for 72 hours with a combination of human-recombinant TGF-β1 0.5 ng/mL (R&D Systems) and IL-1b 2 ng/mL (Peprotech, Rocky Hill, NJ, USA) [64]. The proteins were extracted and were investigated by means of WB as described in Supplementary Table S3.

#### GSK 126 treatment

GSK 126 (Cat. No. S6071, Sellek) was diluted to 5 mM in DMSO and used at doses of 2, 5, 10 and 15 μM. After treatment, the cells were detached using a 1% trypsin-EDTA solution (Cat. No. 15400, Thermo Fischer Scientific), and counted using a standard trypan blue assay. RNA was extracted from the treated and untreated cells, and 500 ng were retro-transcribed to cDNA. SLUG and beta 2 microglobulin cDNAs used as endogenous controls was relatively quantified as described in supplementary materials.

## SUPPLEMENTARY FIGURES AND TABLES


