# Supplementary material for: Epithelioid peritoneal mesothelioma: a hybrid phenotype within a mesenchymal-epithelial/epithelial-mesenchymal transition framework
Source: Oncotarget. 2016 Sep 26;7(46):75503–17. doi: 10.18632/oncotarget.12262 (PMC5342756; doi:10.18632/oncotarget.12262)
Supplement: Supplementary file 1 [file oncotarget-07-75503-s001.pdf]

Epithelioid peritoneal mesothelioma: a hybrid phenotype within a mesenchymal-epithelial/epithelial-mesenchymal transition framework

SUPPLEMENTARY DATA

Patient samples

FISH analyses

FISH analyses were made of all cases except case #19, for which only a limited amount of formalin-fixed, paraffin-embedded material was available. The FISH probes used were LSI CDKN2A Spectrum orange/CEP 9 Spectrum green probes for P16 (Vysis), and RP11-630010 Spectrum green-labelled probes for NF2 (Abbot Molecular), which were used together with spectrum orange-labelled BAC RP11-584A6 mapping to chromosome 3p as the control. The BAC probes were labelled by means of nick translation (Nick Translation Kit, Abbot Molecular) in accordance with the manufacturer's instructions. The results are shown in Supplementary Table S1.

P16 methylation

P16 gene methylation was investigated using specific primers and the protocol published by Herman *et al*. (Proc. Nat. Acad. Sci. USA, Vol 93, pp. 9821-9826, 1996).

MET mRNA in situ hybridisation (ISH)

MET mRNA ISH was carried out as previously described [Cortelazzi *et al*., J Oral Pathol Med., Vol 44, pp. 734-45, 2015]. Briefly, 5 µm sections of formalin-fixed, paraffin-embedded tissue were pre-treated, digested, and hybridised in a hybridiser (Dako Italia S.p.A., Milan, Italy) with the custom-designed MET type-1 probe (Cat. No. va1-10363-01, QuantiGene ViewRNA, Affymetrix, Santa Clara, California, USA) in accordance with the manufacturer's instructions. Positive staining was appeared as red dots in the nucleus and/or cytoplasm. Each run also included the positive control gene peptidylprolyl isomerase B/cyclophilin B (PPIB) type-6 (Cat. No. VA-10509-01, Affymetrix) for evidence of preserved RNA.

VEGFR2 mRNA ISH

VEGFR2 mRNA ISH was carried out manually using the RNAscope kit (Advanced Cell Diagnostics, Inc., Hayward, CA) in accordance with the manufacturer's instructions. In brief, 5 µm sections of formalin-fixed, paraffin-embedded tissue were pre-treated with heat and protease before being hybridised with the Hs-VEGFR2 probe (Cat. No. 312121, Advanced Cell Diagnostics, Inc.). A horseradish peroxidase-based signal amplification system has then bound to the target probes followed by colour development with diaminobenzidine. Positive staining appeared as brown dots in the nucleus and/or cytoplasm. The bacterial gene 4-hydroxy-tetrahydrodipicolinate reductase (DapB) (negative control) and the housekeeping gene ubiquitin C (UbC) (positive control) were included in each run.

miRNA ISH and quantification

MiRNA ISH was carried out as previously described [Gualeni et al, J Clin Pathol. 2015 vol 68, pp661-664 2015]. Briefly, after tissue preparation and permeabilisation, tumoral sections were hybridised for two hours with a double-DIG-LNA probe for hsa-miR-17 (Cat. No. 88084-15, Exiqon, Vedbaek, Denmark) at 61°C, an hsa-miR-21 probe (positive control; Cat. No. 38102-15, Exiqon) at 53°C, and a scramble-miR probe (negative control; Cat. No. 99004-15, Exiqon) at 57°C. Signal detection was automated using the OptiviewDAB Detection Kit (Ventana Medical Systems, Tucson, Arizona, USA) on a Ventana BenchMark ULTRA (Ventana Medical Systems). Positive miRNA 17-5p staining appeared as brown dots usually localised in the cytoplasm. The size and intensity of the dots varied from case to case.

Signals in tumour cells were quantified using a 0-9 scoring system in which the intensity of the signal (0=no signal, 1 = weak, 2 = moderate, and 3 = strong) was multiplied by the percentage of positive cells (0 = negative, 1 = <25%, 2 = 25-50%, and 3 = >50%) [Yang et al, Cancer Cell. vol ;23, pp:186-199, 2013]. Low and high miR-17 expression levels were respectively defined as scores of <6 and ≥6.

Immunofluorescence (IF)

VEGFR2/E-cadherin, E-cadherin/P-cadherin, and E-cadherin/Gata-4 co-expression was investigated by means of IF. The antibodies were diluted as shown in Supplementary Table S2, and the antigen retrieval was carried out using the fully automated BenchMark ULTRA (Ventana) instrument in accordance with the manufacturer's instructions. The slides were incubated with the specific secondary Alexa Fluor antibodies (Alexa Fluor 488 and Alexa Fluor 546, Thermo Fischer Scientific, MA, USA) at room temperature for one hour, and then mounted using Vectashield mounting medium with DAPI (Vector Labs, Burlingame, CA, USA). The samples were observed by means of a Leica DM6000B microscope equipped with a 100 W mercury lamp, with

excitation being obtained using Spectrum Orange (546 nm), Spectrum Green (488 nm) and DAPI excitation filters. The images were acquired through 20x and 40x oil immersion objectives, and analysed using Cytovision software. The images from each channel were collected sequentially in order to limit fluorescence cross-talk. The published images represent extended depth-of-field frames in stack, with focus regions selected on the basis of their maximum intensity.

Biochemistry

The proteins from tissue samples stored at -80°C were homogenised at 4°C in lysis buffer (50 mmol/L HEPES, 150 mmol/L NaCl, 10%glycerol, 1% Triton X-100, 1.5 mmol/L $MgCl_2$, 1 mmol/L EGTA, 10 mmol/L Na_4 P_2O_7, and 100 mmol/L NaF) supplemented with protease and phosphatase inhibitors (Cocktail Inhibitors I and II, Sigma, St. Louis, MO). The lysing involved frequent vortexing, and the lysates were then cleared by means of centrifugation at 13,000 rpm for 30 minutes at 4°C and the proteins were measured using a Bio-Rad protein assay. Western blotting (WB) was carried using standard procedures and 20 µg of proteins. The antibodies used are listed in Supplementary Table S3.

Cell cultures

Fresh aseptic surgical MpM samples were minced and incubated with collagenase (Cat. No. C6885, Sigma) for three hours, and the obtained cell suspension was filtered through a 45 µL nylon mesh, washed with RPMI/FCS 0.5%, diluted in PBS/BSA 1%, and seeded in RPMI (Cat. No. 21875-034, Invitrogen) supplemented with 10% of fetal bovine serum.

Biochemistry

When sub-confluence was reached, the cells were detached as described above for the surgical samples, and analysed by means of WB using the antibodies and conditions shown in Supplementary Table S3.

Flow cytometry (FC)

FC staining was used in order to distinguish MpM and non-MpM cells in the primary cultures. Cells obtained after detachment with trypsin-EDTA (Cat. No. 15400, Invitrogen) were washed twice in PBS, counted, and diluted to an appropriate concentration for staining with CAM 5.2 FITC (Cat. No. 35303, Becton Dikinson). Briefly, $2x10^5$ cells were placed in a 12 x 75 mm round-bottomed polystyrene tube and, after washing with PBS/BSA, the cells were labelled with CAM 5.2 FITC using the Fix and Perm Kit (Cat. No. GAS001, Invitrogen) in accordance with the manufacturer's instructions. At least $5x10^4$ cells were acquired using a FACS-Canto 2 cytometer, and analysed using FACS DIVA or Win MDI software.

Immunoistochemistry (IHC)

The primary and stabilised cell cultures were detached using trypsin, and fixed for one hour at room temperature in a 10% solution of buffered formalin. Formalin-fixed, paraffin-embedded (FFPE) cell blocks suitable for IHC were then obtained using standard procedures. The IHC experiments were performed under the conditions shown in Supplementary Table 2.

RNA analyses

RNA was extracted from the cells using an RNeasy minikit (Cat. No. 74104, Qiagen), and quantified using nano-drop device. Five hundred nanograms of of RNA was used for RT-PCR and cDNA synthesis. cDNA retro-transcribed from 500 ng of RNA obtained from U-87 glioma cell line was used as a reference sample.

Real-time quantitative PCR

CD90, Axl, Alcam, Zeb1, CD105, c-Myc, Slug, Twist, Erb2 and beta 2 microglobulin were relatively quantified by means of real-time quantitative PCR (ABIPRISM 5700 PCR, Applied Biosystems), and the $2^{-\Delta\Delta Ct}$method was used to calculate the relative changes in gene expression. All of the probes came from Thermofisher Scientific. CD90 (Hs 00264235_m1), Axl (Hs 1064444_m1), Alcam (Hs 00977641_m1), Zeb1 (Hs 00232783_m1), CD105 (Hs 00923996_m1), c-Myc(Hs00153408_m1), Slug (Hs 00 950344_m1), Twist (Hs 02379973_m1), Erb2 (Hs 00170433_m1) and beta 2 microglobulin (Hs00187842_m1)

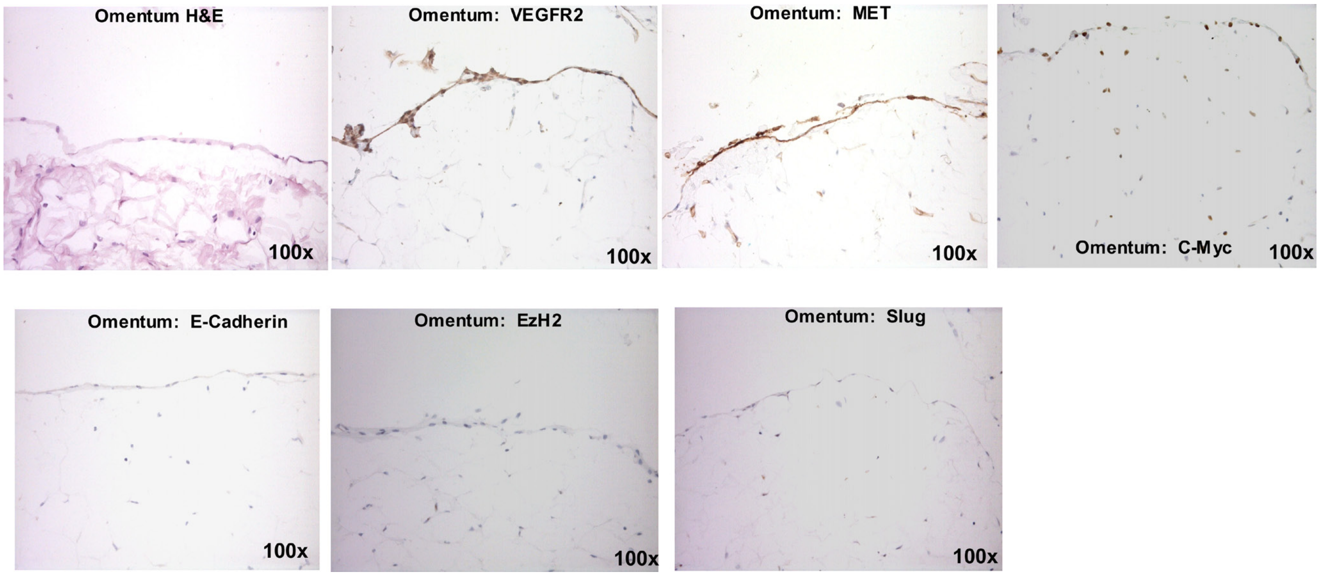

Supplementary Figure S1: Mesothelium IHC profile. IHC was performed using three formalin-fixed, paraffin-embedded omentum samples obtained from unrelated MpM patients. The IHC conditions are shown in Supplementary Table S2. The microphotographs show the VEGFR2-, c-MET- and c-MYC-positive, and E-cadherin-, SLUG and EZH2-negative mesothelium immunophenotype. Original magnifications: 100X (as indicated in each figure).

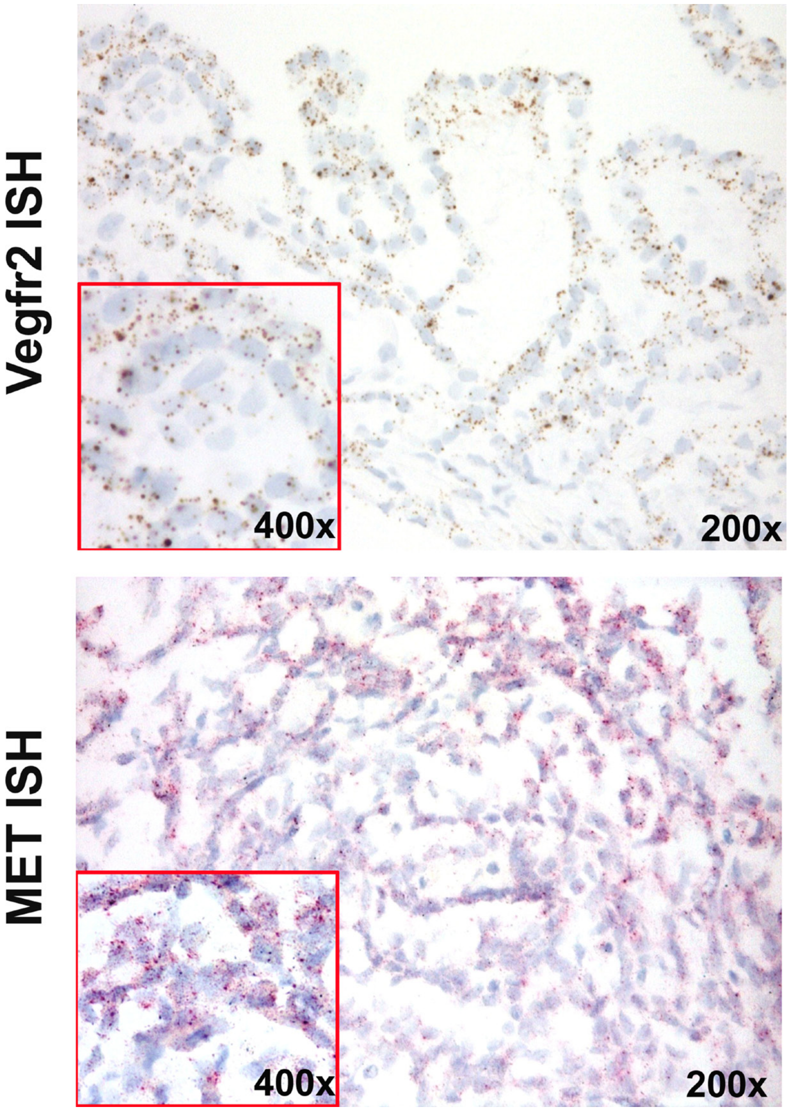

Supplementary Figure S2: VEGFR2 RNA and MET RNA ISH. Both pictures show the large number of dot-like signals restricted to epithelioid tumoral cells in a case of E-MpM (E-MpM1, Table 1). Original magnifications: 200X and 400x (inset).

Case #16, Table 1

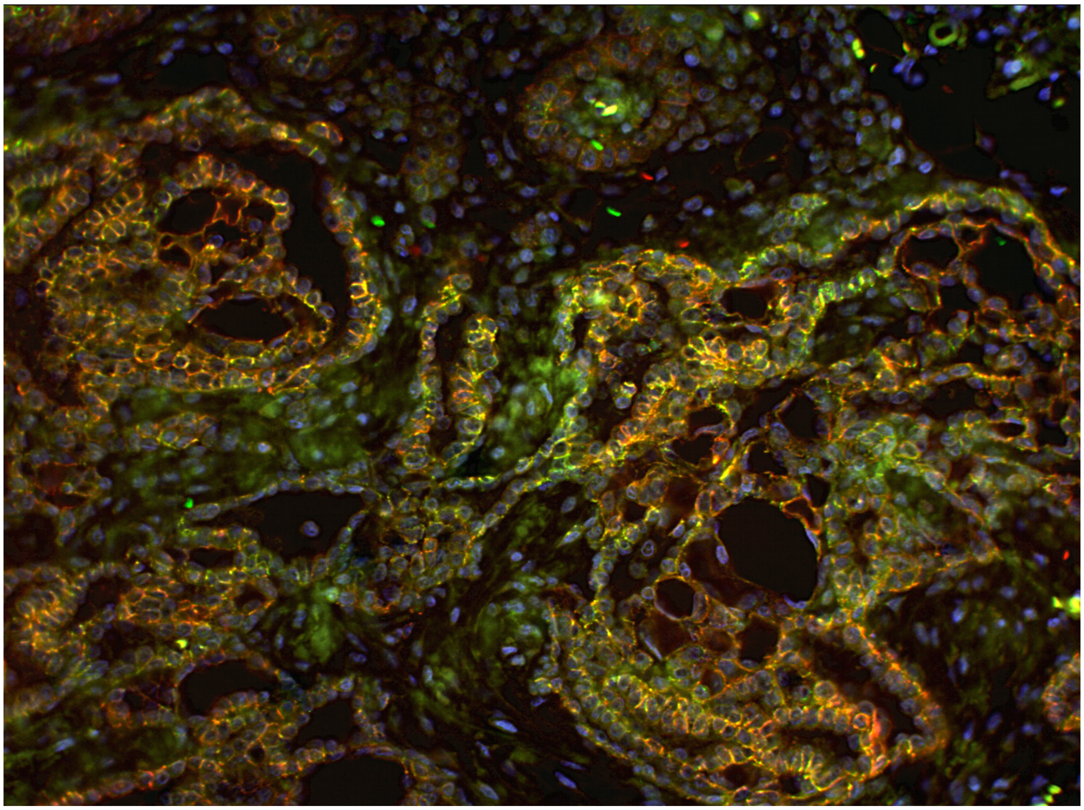

Supplementary Figure S3: E-cadherin-VEGFR2 Immunofluorescence. E-cadherin (red) and VEGFR2 (green) immunofluorescence highlights the hybrid phenotype and the heterogeneity of E-MpM (#16, Table 1). Original magnifications: 40X.

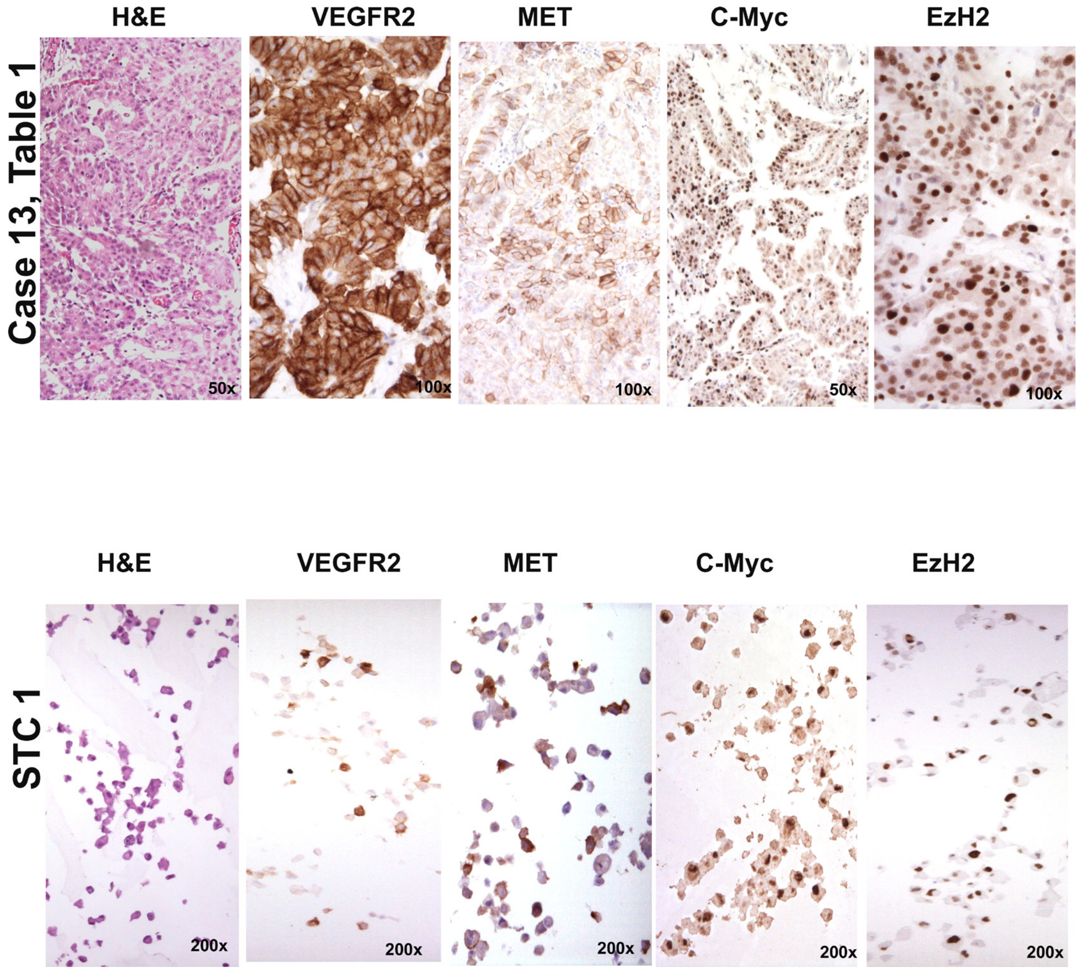

Supplementary Figure S4: Comparison of the VEGFR2, MET, c-MYC, EZH2 immunoprofiles of the E-MpM surgical samples (#11, Table 1) and its derived STC 1. Formalin-fixed, paraffin-embedded cell blocks suitable for IHC were obtained after trypsin cell detachment. IHC was performed under the conditions shown in Supplementary Table S2. The image gallery compares the VEGFR2, MET, c-Myc and EZH2 immunoprofiles of three E-MpM surgical specimens with those of their derived cell lines. The two profiles show optimal compliance. Original magnifications: 50X, 100X and 200X (as indicated in each figure).

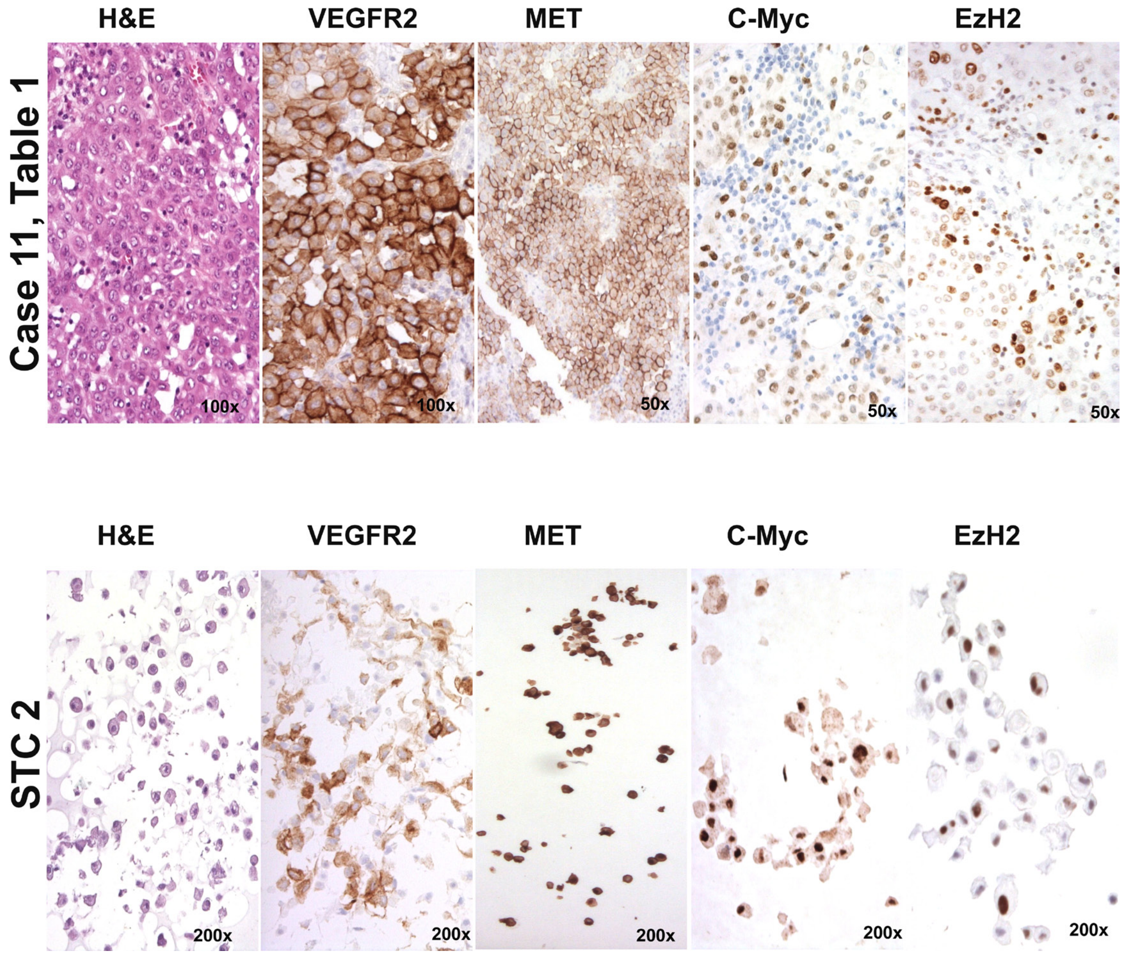

Supplementary Figure S5: Comparison of the VEGFR2, MET, c-MYC, EZH2 immunoprofiles of the E-MpM surgical samples (#13, Table 1) and its derived STC2. Formalin-fixed, paraffin-embedded cell blocks suitable for IHC were obtained after trypsin cell detachment. IHC was performed under the conditions shown in Supplementary Table S2. The image gallery compares the VEGFR2, MET, c-Myc and EZH2 immunoprofiles of three E-MpM surgical specimens with those of their derived cell lines. The profile shows optimal compliance. Original magnifications: 50X, 100X and 200X (as indicated in each figure).

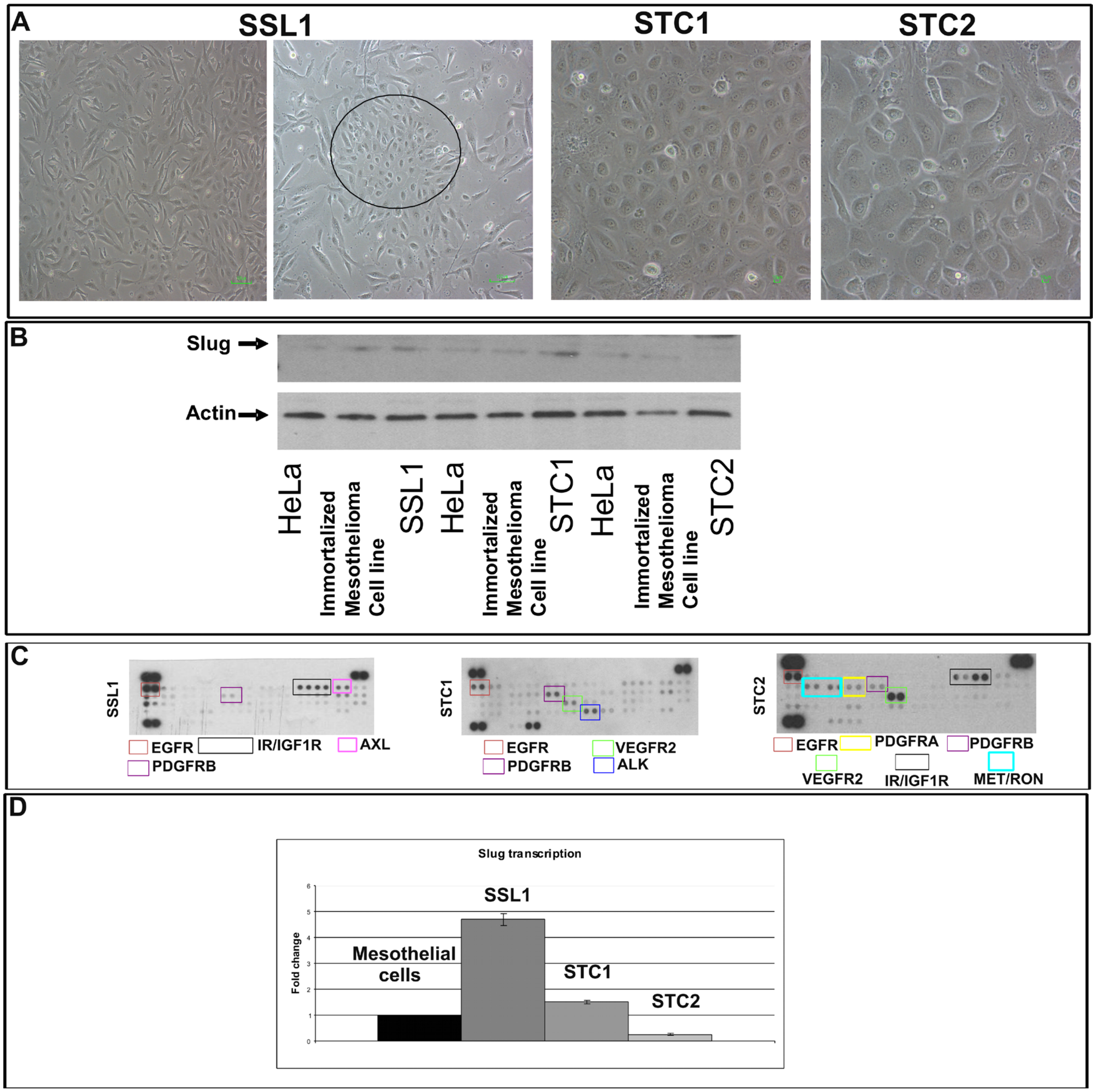

Supplementary Figure S6: Morphology, SLUG expression, VEGFR2 activation and slug transcription rate. A. SSL1 mainly consisted of spindle-shaped cells that were able to acquire a more epithelioid morphology spontaneously (black circle), whereas STC1 and STC2 consisted of round cells that were larger in STC2 than STC1. **B.** Slug expression was detected by WB in SSL1 and STC1, but not in STC2. HeLa and one mesothelioma Htert-immortalised cell line were used as positive controls. **C.** Receptor tyrosine kinase activation profiles were investigated using a phospho-RTK array kit (Proteome Profiler™ Array, ARY001B, R&D Systems): the activated receptors are boxed and indicated. **D.** In line with the Western blot findings, SSL1 had the highest slug transcription rate. Interestingly, STC2 also had a lower transcription rate than one omentum-derived mesothelial cell used as a reference. These characteristics suggest that, in comparison with their corresponding clinical samples (all epithelioid mesotheliomas), mesothelioma cells grown *in vitro* undergo incomplete EMT. SSL1 original magnification 20x; scale bar 10 µM; STC1 and 2 original magnifications 10x; scale bar 100 µM.

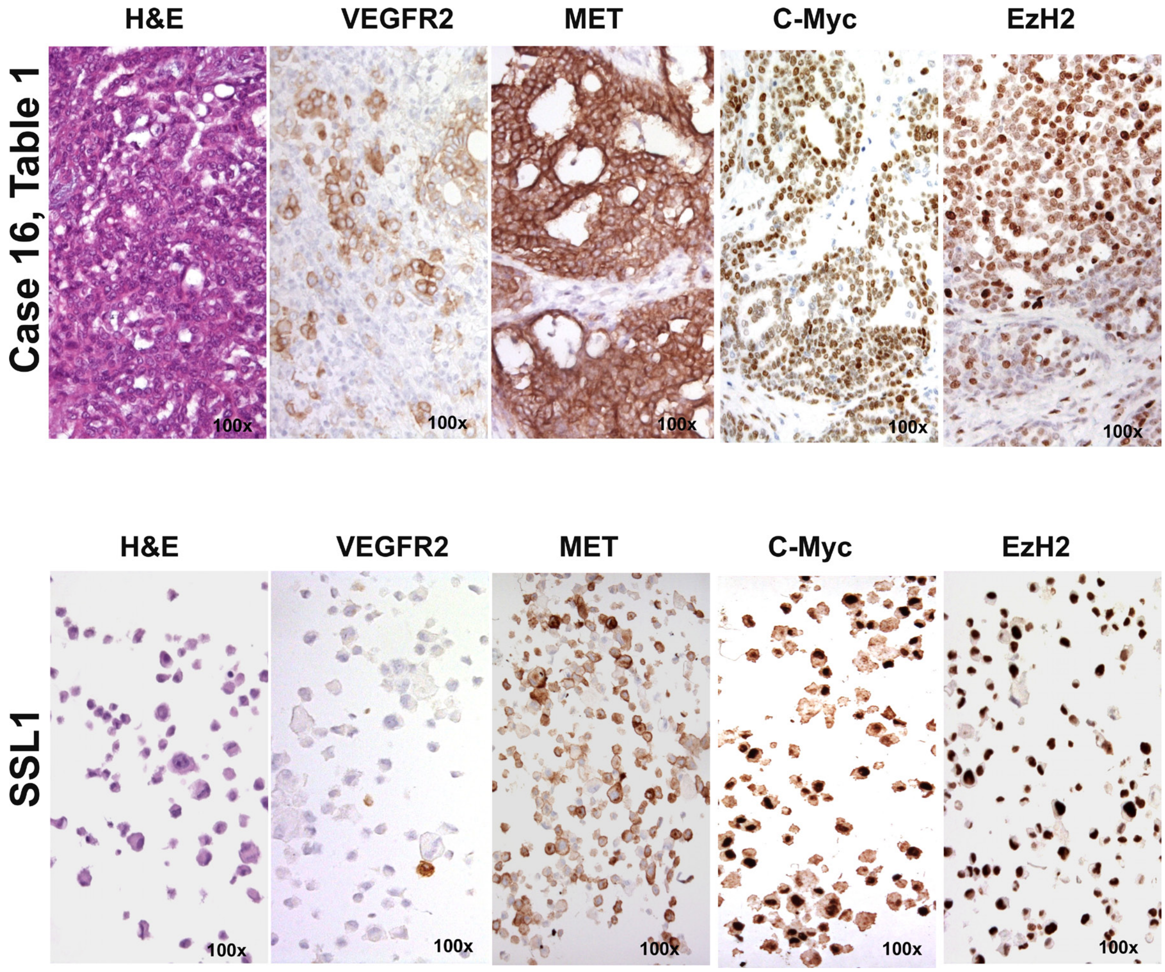

Supplementary Figure S7: Comparison of the VEGFR2, MET, c-MYC, EZH2 immunoprofiles of the E-MpM surgical sample E-MpM (#16, Table 1) and its derived cell line (SSL1). Formalin-fixed, paraffin-embedded cell blocks suitable for IHC were obtained after trypsin cell detachment. IHC was performed under the conditions shown in Supplementary Table S2. The image gallery compares the VEGFR2, MET, c-Myc and EZH2 immunoprofiles of E-MpM #16 surgical specimens with those of its derived cell lines. The profile shows optimal compliance. Original magnifications:100X and 200X (as indicated in each figure).

Supplementary Table S1: Methylation, gene status and/or protein expression of p16, NF2 and BAP-1

		p16			NF2	BAP1	
		**Met**	**FISH**	**IHC**	**FISH**	**IHC**	
**#1**	E-MPM 1	Np	Nor	-	Nor	+	p16
**#2**	E-MPM 2	Np	Omo	-	Emi	-	p16/bap1
**#3**	E-MPM 3	Np	Nor	-	Nor	-	P16/Bap1
**#4**	E-MPM 4	M	Emi	-	Emi	-	P16/bap1
**#5**	E-MPM 5	NV	Emi	+	Emi	-	Bap1
**#6**	E-MPM 6	M	Emi	-	Omo	-	P16/NF2/bap1
**#7**	E-MPM 7	NP	Omo	-	Nor	-	P16/bap1
**#8**	E-MPM 8	U	Nor	-	Nor	+	P16
**#9**	E-MPM 9	M	Nor	+*	Nor	+	P16
**#10**	E-MPM 10	NV	Emi	+*	Omo	-	Bap1/NF2
**#11**	E-MPM 11	U	Nor	+*	Nor	-	Bap1
**#12**	E-MPM 12	M	Emi	-	Emi	+	P16
**#13**	E-MPM 13	NP	Emi	-	Emi	+	P16
**#14**	E-MPM 14	NP	Omo	-	Emi	+	P16
**#15**	E-MPM 15	NP	Omo	-	Nor	-	P16/bap1
**#16**	E-MPM 16 progressed	M	Nor	-	Emi	-	P16/bap1
**#17**	E-MPM 17 progressed	NP	Omo	-	Emi	NV	P16
**#18**	HG E-MPM 1	M	Emi	+*	Nor	+	P16
**#19**	HG E-MPM 2	Np	Np	-	NP	NP	P16
**#20**	S-MPM 3	Np	Omo	-	Nor	+	P16
**#21**	S-MPM4	M	Nor	+*	Nor	+	P16

p16 inactivation was investigated by promoter methylation (11 out of 21 cases), FISH (20 out of 21 cases) and IHC (all cases). NF2 inactivation was performed by FISH in 20 out of 21 cases. BAP-1 IHC was performed in 20 out of 21 cases.
The most frequently deleted gene was p16 (18 cases) followed by BAP-1 (10 cases) and NF2 (2cases). In blue are highlighted cases that showed only p16 deletion (11 cases), in yellow cases (6 cases) that showed p16 and BAP1 inactivation and 1 cases had p16, Bap1 and NF2 deletions (purple). Two cases showed only BAP1 inactivation (green) and 1 case had Bap 1 plus NF2 inactivation (grey). Collectively more than 85% of the specimens had surpassed senescence; the rest showed deregulation of the tumour suppressor gene and chromatin regulator BAP-1 (Liao L, Testa JR, Yang H. The roles of chromatin-remodelers and epigenetic modifiers in kidney cancer. Cancer Genet. 2015;208:206-214).
The following FISH probes were employed:
P16: LSI CDKN2A Spectrum orange/CEP 9 Spectrum green probes (Vysis);
NF2: RP11-630010 Spectrum green labelled (Abbot molecular)
BAP1: BAC RP11-630010 Spectrum green labelled (Abbot molecular) used alongside with spectrum orange labelled BAC RP11-584A6 mapping on chromosome 3p as a control.
BAC probes were labelled by nick translation (nick translation kit, Abbot molecular) according with manufacturer's instructions.
Met: p16 gene promoter methylation (detected by PCR);
IHC: immunohistochemistry;
Nor: Normal disomic pattern;
Omo: homozigous deletion;
Emi: hemizigous deletion;
NP: not performed;
NV: not evaluable
+*: samples showing cytoplamatic immunoreactivity in tumoral cells.

Supplementary Table S2: Antibodies used, source and Immunohistochemical conditions

Antibody	Cat. No.	Company	Dilution	Antigen retrieval/Detection	Positive control
Bap1	Sc-28383	Santa Cruz	1:100	Benchmark ULTRA Ventana, extended, CC1 buffer, optiview, DAB	Normal pancreas
p16	805-4713	Cintec	Prediluted	Benchmark ULTRA Ventana, extended	Squamous cell carcinoma
INI1	612111	BD Transduction	1:200	Autostainer Link 48 (Dako) EDTA 30' + Linker mouse	Nephroblastoma
VEGFR2	2479S	Cell Signaling	1:300	Benchmark ULTRA Ventana, extended, CC1 buffer, ultraview, DAB.	Angiosarcoma
c-MET (clone SP-44)	790-4430	Ventana	1:50	Benchmark ULTRA Ventana, extended, CC1 buffer, ultraview, DAB.	Clear cell sarcoma
E-cadherin	M3612	Dako	1:50	Benchmark ULTRA Ventana, extended, CC1 buffer, ultraview, DAB.	Colorectal cancer
C-MYC	Ab 37072	Abcam	1:50	Benchmark ULTRA Ventana, extended, CC1 buffer, ultraview, DAB.	Round cell myxoid liposarcoma
EZH2	5246S	Cell Signaling	1:100	Benchmark ULTRA Ventana, extended, CC1 buffer, ultraview, DAB.	Breast cancer
Mib1	M7240	Dako	1:400	Autostainer Link 48 (Dako) EDTA 15' + Linker mouse	Colorectal cancer
SLUG	9585S	Cell Signaling	1:50	Benchmark ULTRA Ventana, extended, CC1 buffer, optiwiev, DAB.	Solitary fibrous tumour
Twist	Sc-81417	Santa Cruz	1:50	Benchmark ULTRA Ventana, extended, CC1 buffer, optiwiev, DAB	Solitary fibrous tumour
P-cadherin	2130S	Cell Signaling	1:50	Benchmark ULTRA Ventana, extended	Kidney
Beta catenin	610154	BD Transduction	1:1000	Autostainer Link 48 (Dako) EDTA 15' + Linker mouse	Colorectal cancer
Smooth muscle actin (clone 1A4)	M0851	Dako	1:100	Benchmark ULTRA Ventana, extended	GIST
GATA4	Sc-25310		1:100	Benchmark ULTRA Ventana, extended	Brest cancer

A representative paraffin block of formalin-fixed tumoral tissue was selected and phenotyped for each case.
The antibodies were diluted as shown in the table; antigen retrieval and development were carried out using the fully automated benchmark ULTRA (Ventana) or Autostainer Link 48 (Dako) instruments in accordance with the manufacturers' instructions.

Supplementary Table S3: Antibodies and Western blot conditions

Antibody	Catalogue	Company	Dilution	Positive control
Anti-pTyr	05-321	Millipore	1:3000	
MET y1230-34-35	Ab 5662	Abcam	1:1000	A431 cell line
MET	M3440	Spring	1:1000	A431 cell line
E-cadherin	M3612	Dako	1:1000	A431 cell line
c-MYC	Ab 37072	Abcam	1:5000	HeLa cell line
EZH2	5246S	Cell Signaling	1:1000	HeLa cell line
SLUG	9585S	Cell Signaling	1:1000	HeLa cell line, Htert immortalised mesothelioma cell line
TWIST	Sc-81417	Santa Cruz	1:500	HeLa cell line, Htert immortalised mesothelioma cell line
ACTIN	A 2066	Sigma	1:4000	
